# Legal but lethal: functional protein aggregation at the verge of toxicity

**DOI:** 10.3389/fncel.2015.00045

**Published:** 2015-02-18

**Authors:** Angelika Falsone, S. Fabio Falsone

**Affiliations:** Institute of Pharmaceutical Sciences, University of GrazGraz, Austria

**Keywords:** amyloids, prions, proteotoxicity, neurodegenerative diseases, proteostasis regulators

## Abstract

Many neurodegenerative disorders are linked to irreversible protein aggregation, a process that usually comes along with toxicity and serious cellular damage. However, it is emerging that protein aggregation can also serve for physiological purposes, as impressively shown for prions. While the aggregation of this protein family was initially considered exclusively toxic in mammalians organisms, it is now almost clear that many other proteins adopt prion-like attributes to rationally polymerize into higher order complexes with organized physiologic roles. This implies that cells can tolerate at least in some measure the accumulation of inherently dangerous protein aggregates for functional profit. This review summarizes currently known strategies that living organisms adopt to preserve beneficial aggregation, and to prevent the catastrophic accumulation of toxic aggregates that frequently accompany neurodegeneration.

## Introduction

Low structural complexity is at the basis of highly diversified molecular recognition, whereby one flexible protein region can bind to various heterogeneous ligands by conformational adaptation. Proteins can functionally benefit from binding promiscuity for key regulatory processes such as signal transduction, transcription, RNA processing and translation. Proteins situated on intersecting hubs of different pathways can undergo multifunctional interactions, functioning as molecular switches by means of conformational variability. However, the benefits of conformational freedom come along with the menace of protein misfolding and multifunctional failure. Although each living organism invests conspicuous amounts of energy for the rescue or elimination of misfolded polypeptides, a possible inability of the cell to cope with misfolded polypeptides inevitably leads to a massive functional destabilization, whereby proteins can either lose their original function (loss-of-function), or they acquire an improper, and therefore mostly lethal function (gain-of-function), eventually aggregating after the complete collapse of folding and clearance pathways.

The central nervous system (CNS) is particularly susceptible to protein misfolding, but the reasons of such selective neuronal vulnerability are still elusive (Saxena and Caroni, 2011). Although healthy adult neurons can manage proteostasis by standard folding and degradation routines (see Section Molecular catchers in the amyloid rye: heat shock proteins and proteolytic pathways), macroscopic protein misfolding manifestations prevail with senescence, as attested by the occurrence of ageing-associated neurodegenerative disorders such as Parkinson Disease (PD), Alzheimer Disease (AD), spongiphorm encephalopathies, or amyotrophic lateral sclerosis (ALS), all of them displaying unsoluble protein inclusions as a signature.

## Amyloid aggregation

### Structural properties of amyloids

Irrespectively of the protein structure and the exact anatomic localization, one type of proteinaceous inclusions appearing in the CNS features some unifying histologic and biophysical hallmarks classified under the term “amyloid”, consisting of (A) a fibrous, non-branched morphology, (B) the ability to alter the spectral properties of the dyes Congo red and thioflavin T, and (C) X-ray diffraction patterns typical of cross-beta structure. The term was coined by German pathologist Rudolf Virchow during the characterization of masses in human brains described as “corpora amylacea”. Virchow perceived the relationship between amyloids and disease, when he addressed the problem of “amyloid degeneration” (Virchow, 1855). Today, it is well established that the appearance of amyloids is associated with a chronic tissue degeneration of the brain, and although not restricted to this organ, our current understanding of misfolding diseases is invariably linked to amyloid-associated neurological brain disorders (For a detailed list of known amyloid diseases, we refer to the classification of the International Society of Amyloidosis Sipe et al., 2014).

Amyloid aggregation is stereotypically linked to conformational flexibility, which allows for structurally diverse polypeptides to fold from a native into an alternative structure of the same chemical composition, but with high beta-sheet content, a remarkable resistance against proteolytic and denaturing agents, and the ability to self-associate into fibers of typical cross-beta structure (Eisenberg and Jucker, 2012). The pathway of this association is dynamically variegated, highly perturbable, and sensitive to a repertoire of factors such as genetic mutations, small ligands, changes of the physico-chemical environment, or post-translational side chain modifications (Eichner and Radford, 2011). The resulting heterogeneous multiplicity of conformational intermediates differing in size, shape and stability poses an obstacle in isolating and unambiguously categorizing amyloid aggregates, especially these conformers among them which are believed to be proteotoxic (Eisenberg and Jucker, 2012), and which likely accumulate in low amounts somewhere between native polypeptide and mature amyloid. Improved biophysical techniques can in part overcome these limitations, and they are beginning to provide useful structural insights of amyloid folding intermediates. By combining rapid fluorescence techniques with NMR spectroscopy, Sarkar et al. measured conformational fluctuations of a short-lived, low-abundance AD-associated beta-amyloid (Abeta) oligomer (Sarkar et al., 2014). They could pinpoint the dynamic structure of this oligomer to a patchwork of amino acid segments which fold locally before the transition into highly ordered amyloid filaments. Likewise, Röthlein et al. employed time resolved fluorescence and computational calculations to obtain structural views of an extremely unstable huntingtin (htt) amyloid filament (Röthlein et al., 2014).

### Molecular pathways of amyloid propagation

Amyloid folding has been coupled early to prion diseases (Prusiner et al., 1983), a type of devastating neurodegenerative disorders that are transmitted by direct and self-sustained intercellular propagation of toxic agents, which constitutes an outstanding and devastating strategy to bypass genetic routes of infection. While the infectious prion particle known as PrP^Sc^ is typically amyloid (Prusiner et al., 1983), the cellular conformer PrP^c^ is a non-amyloid, non-infectious globular protein of still undefined function. This striking difference led to suppose that prion proteins must access an amyloid state to acquire transmissible properties.

A growing amount of studies suggests that this type of cell-to-cell transmission is apparently common to amyloids, as it has been observed also for other amyloidogenic proteins with well-known implication in neurodegenerative diseases (Table 1), suggesting that the amyloid fold is a structural prerequisite of cell-to-cell propagation (Eisenberg and Jucker, 2012). Indeed, it was discovered, that amyloidogenic proteins such as alpha-Synunclein (aSyn), tau, or Abeta can also propagate between cells, and that these transmissible particles share structural properties closely similar to those of the archetypic infectious particle PrP^sc^: they have a predominant beta-sheet structure, a remarkable resistance against proteolytic and denaturing agents, and a self-templating ability (Prusiner et al., 1983; Luk et al., 2012; Iba et al., 2013; Sanders et al., 2014; Stöhr et al., 2014; Watts et al., 2014). Similar rearrangements are likely to occur for endogenous tau in mice brains upon the stereotactic delivery of toxic tau seeds, which is sufficient to initiate spreading of AD-like neurofibrillary tangles along a spatially defined trajectory (Iba et al., 2013). In close analogy, the injection of aSyn seeds into brains of healthy mice triggers the self-perpetuating polymerization of endogenous aSyn and the development of clinical symptoms of PD (Luk et al., 2012). In both cases, the sequential diffusion of proteotoxicity along interconnected brain regions substantiates the theory of a staged evolution of PD and AD (Braak et al., 2006). With respect to original prions, all these proteins have thus been attributed “prion-like” or “prionoid” propagation properties. However, complicating a pathologic interpretation is the occurrence of different conformationally variable toxic species from the same protein, with separate propagation behavior and distinct phenotypic manifestations of the same condition. Sanders et al. demonstrated that tau, the signature protein of tauopathies, forms biochemically and morphologically distinct oligomers accounting for different disease phenotypes, with AD inclusions showing the most homogenous composition (Sanders et al., 2014). Similar has been shown for Abeta, the major component of AD amyloid plaques. Different patient-derived as well as synthetic species of Abeta display an individual transmission behavior, accumulation pattern, aggregate morphology, and chemical stability (Stöhr et al., 2014; Watts et al., 2014). Bousset et al., also reported of two differently toxic aSyn conformers with separate propagation properties (Bousset et al., 2013). Additional complexity rises from the ability of particular amyloid aggregates to act as cross-nucleation seeds for other structurally unrelated proteins. Alpha-Synunclein (aSyn), the major constituent of PD associated Lewy Inclusions, can initiate the deposition of tau in primary neurons and transgenic mice (Guo et al., 2013), while TDP-43 amyloids can seed for Abeta fibril growth (Fang et al., 2014), which might contribute to co-morbidities observed for some types of amyloid pathologies.

**Table 1:** **Summary of representative proteins handled within this review, with respect to pathology, and prion/prion-like properties**.

Protein name	Most relevant associated diseases	Amyloid polymerization	Cell-to-cell transmission	Exosomal secretion	HSPG binding
aSyn	PD;	Yes^a^	Yes (Luk et al., 2012)	Yes (Alvarez-Erviti et al., 2011)	Yes (Holmes et al., 2013)
	Dementia with Lewy Bodies
Abeta	AD	Yes^a^	Yes (Stöhr et al., 2014; Watts et al., 2014)	Yes (Rajendran et al., 2006)	Yes (Holmes et al., 2013)
PrP	spongiphorm encephalopathies	Yes^a^	Yes^a^	Yes (Fevrier et al., 2004)	Yes (Horonchik et al., 2005)
tau	tauopathies	Yes^b^ (Iba et al., 2013; Morozova et al., 2013)	Yes (Iba et al., 2013)	Yes (Saman et al., 2012)	Yes (Holmes et al., 2013)
FUS	ALS; FTD	Yes (Schwartz et al., 2013 (in the presence of RNA); Nomura et al., 2014 (G156E variant); Han et al., 2012; Kato et al., 2012; Kwon et al., 2013 (FUS low complexity region)).	?	?	?
TDP-43	ALS; FTD	Yes (Guo et al., 2011; Fang et al., 2014 (A315T variant); Chen et al., 2010; Furukawa et al., 2011 (G294A variant))	Yes (Nonaka et al., 2013)	Probable (Nonaka et al., 2013)	?
Cu/Zn SOD	ALS	Yes (DiDonato et al., 2003(ALS-associated mutants))	Yes (Münch et al., 2011; Grad et al., 2014)	Yes (Grad et al., 2014)	Yes (Inoue et al., 1991)
hnRNPA	multiple system proteinopathy	Yes (Kato et al., 2012; Kim et al., 2013)	Yes (Kato et al., 2012; Kim et al., 2013)	?	?
TIA-1	Welander distal myopathy	Yes (Furukawa et al., 2009; Li et al., 2014)	Yes (Li et al., 2014)	?	?
polyQ-huntingtin (htt)	HD	Yes (Lotz et al., 2010; Falsone et al., 2012)	?^c^	?	No (Holmes et al., 2013)
CPEB^d^	-	Yes (Raveendra et al., 2013)	Yes (Si et al., 2003)	?	?
Sup35^e^	-	Yes^a^	Yes^a^	?	-

^a^prototypic representatives

^b^in the presence of heparin

^c^prion-like propagation with synthetic polyQ fibrils (Ren et al., 2009)

^d^from Aplysia californica

*^e^from Saccharomyces cerevisiae*.

#### Exosomes

We have only a slight clue on the mechanisms of intercellular prion spreading. Recent investigations point to exosomes (Table 1), a type of membrane-enclosed vesicle sized 30–100 nm, with the ability of transporting a remarkably diverse cargo ranging from active proteins to different RNA particles for cellular exchange (Kowal et al., 2014). Exosomes have been associated with prion transmission since the isolation of PrP^sc^ from exosome preparations after cellular infection with sheep prions (Fevrier et al., 2004), and PrP-loaded exosomes could be successively isolated from brain fluids of infected animals (Vella et al., 2008).

It seems that the exosome-mediated secretion of amyloid-associated proteins is an organized process, as it can be triggered by determinate signaling events, such as calcium release (Emmanouilidou et al., 2010), or platelet activation (Robertson et al., 2006). Of fundamental importance, PrP^sc^ or aSyn oligomers isolated from exosomes retain full toxicity, suggesting that these organelles can indeed serve as infectious vehicles (Fevrier et al., 2004; Vella et al., 2007; Danzer et al., 2012). This leads to hypothesize that the exosome-mediated release of noxious agents might originally represent a rational strategy to relieve cells from toxicity. Such a hypothesis is supported by studies showing that when the intracellular clearance of aSyn is compromised, an elevated deployment of exosomes carrying this protein as a cargo becomes observable (Alvarez-Erviti et al., 2011; Danzer et al., 2012). With this respect, an intact exosomal secretion pathway appears essential for neuroprotection. Upon the manipulation of PARK9/ATP13A2, a component of the exosome biogenesis machinery, cells can less efficiently counteract intraneuronal aSyn toxicity. Accordingly, surviving neurons from PD patients display increased levels of PARK9/ATP13A2 (Kong et al., 2014), whereas affected neurons show decreased levels (Murphy et al., 2013). In a purely speculative way, deregulated cells that are no longer able to efficiently handle the clearance of proteotoxic species might pass over their own toxic burden to functioning cells for disposal.

Interestingly, *in vitro* generated aSyn, tau, Abeta, PrP or polyQ aggregates can be efficiently internalized when directly applied to growing cells or animals, apparently without an exosomal delivery (Horonchik et al., 2005; Ren et al., 2009; Luk et al., 2012; Holmes et al., 2013; Iba et al., 2013; Aulić et al., 2014; Volpicelli-Daley et al., 2014), suggesting the existence of additional pathways of transmission. As shown for ALS-associated Cu/Zn-superoxide dismutase (SOD), parallel exosome dependent and independent mechanisms have been postulated, whereby an active exosome-associated secretion can be backed by a passive, carrier-free diffusion of toxic particles, probably upon their release from necrotizing cells (Grad et al., 2014).

#### Proteoglycans

A recent study identifies heparan sulphate proteoglycans (HSPGs) as further key players of intercellular transmission (Holmes et al., 2013; Table 1). HSPGs are a class of membrane proteins conjugated to the heavily sulphated glycosaminoglycan (GAG) heparan sulphate, which is a constituent of the extracellular matrix (Xu and Esko, 2014). Although HSPGs are traditionally coupled to cell attraction and migration, a pathologic link with amyloid diseases has been postulated ever since the identification of GAGs in various types of amyloid deposits from affected brains (Snow et al., 1988, 1990; Spillantini et al., 1999).

HSPGs are direct targets of pathologic PrP, aSyn, Abeta and tau (Horonchik et al., 2005; Holmes et al., 2013). All of them become actively internalized after HSPG binding, consequently propagate within neuronal cells, and sustain the development of a pathologic condition. The physico-chemical integrity of the GAG-chains appears essential for binding, as the subsequent internalization becomes affected upon chemical or genetic alteration of the GAG residues (Holmes et al., 2013). Indeed, GAGs were attributed amyloid modifying properties *in vitro* depending on the size (Vieira et al., 2014) and charge (Lawson et al., 2010) of the GAG chain. Given that the proteoglycan expression and GAG-composition are cell-specific and vary with brain development and senescence (Rykova et al., 2011), it can be speculated that ageing-related changes in cell-surface proteoglycan patterns will influence HSPG-mediated prion-like propagation.

HSPGs might represent a converging hub for exosome-dependent and independent prion propagation, owing to some potential overlaps between both pathways. HSPGs can regulate also exosome internalization (Christianson et al., 2013), and GAG modifications affect both amyloid internalization and exosomal uptake (Christianson et al., 2013; Holmes et al., 2013). Both processes further initiate macropinocytosis, a mechanism whereby macromolecules are taken up by actin-membrane ruffles (Fitzner et al., 2011; Holmes et al., 2013). Finally, exosomes and proteopathic seeds activate identical neuroinflammatory pathways (Thellung et al., 2007; Tomasi, 2010; Christianson et al., 2013). Collectively, these considerations suggest an exosome/HSPG unifying route for prion entry.

## Amyloid function

### Physiologic significance of amyloid polymerization

While in humans the concept of amyloid has been traditionally interpreted in terms of lethality, observations from various evolutionary distinct organisms strongly support a role that extends well beyond toxicity. In prokaryotes, cell-surface amyloid polymerization is quite diffuse (Dueholm et al., 2013), and the biogenesis of curli filaments from enterobacteria is an example of how unicellular organisms use amyloidogenesis for physiological processes such as biofilm formation, host adhesion, and cellular clustering. CsgA protein, the principal component of curli in *E. coli*, polymerizes into amyloids after the secretion across the outer membrane. *In vivo*, this process is strictly regulated by CsgB, an accessory protein whose gene is localized to the same operon as the *CsgA* gene, and that serves as an obligatory nucleation seed for CsgA polymerization (Shu et al., 2012). CsgB rapidly assembles into beta-sheet rich oligomers that massively catalyze the transition of inherently unstructured CsgA monomers into amyloid. Interestingly, *in vitro* isolated CsgA can separately polymerize into amyloid fibrils also in the absence of CsgB. This difference suggests that curli amyloidogenesis in living cells is under the stringent control of a dedicated trigger which actually restricts the assembly of amyloids to an immediate physiological request.

In mammalian organisms, a functionally controlled amyloid growth process has been proposed for melanosomes, a type of organelles used for the synthesis and storage of melanin body pigments. The transmembrane protein Pmel17 was identified as a major component of amyloid fibers that organize in linear arrays through the length of the organelle (Raposo et al., 2001), and functionally serve as melanin deposits. Pmel17 is a multidomain protein that undergoes multiple sequential posttranslatory processing and protease cleavage steps on its way to becoming functionally incorporated into amyloid fibers on the membrane of mature melanosomes (Leonhardt et al., 2013). One critical point is the late cleavage and extralumenal release of the N-terminally located regions RPT (an imperfect repeat region), PKD (polycstic kidney disease domain), and NTR (N-terminal region). RPT and PKD represent soluble core amyloidogenic fragments that can assemble into fibers once cleaved, while NTR is not amyloidogenic, and seems to serve as a specific stabilizer of RPT and PKD during fibril growth. When this property of NTR is impaired, the fibrillogenic fragments are degraded and amyloid polymerization of RPT and PKD cannot occur.

Both mechanisms described above highlight a possible safeguard strategy by which the integrated generation of specific amyloid promoting factors simultaneously to amyloidogenic particles drives polymerization towards mature amyloid fibers instead of potentially harmful misfolded intermediates. This provides a means to exploiting stringently controlled amyloid polymerization for functional purposes.

One further example for function-associated amyloidogenesis comes from mammalian secretory granules of pituitary glands, where a set of peptide hormones accumulate in form of aggregates with amyloid properties, being reactive to amyloid-specific immunodetection, ThioS and Congo Red staining, and with a typical cross-beta x-ray diffraction pattern (Maji et al., 2009). Interestingly, a variety of isolated peptide hormones is capable of amyloid fiber formation at moderately acidic pH and in the presence of glycosaminogylcans, two conditions similar to those within secretory granules, while partially resolubilising when exposed to extragranular conditions (pH 7.4). This behavior leads to suggest that secretory granules can store hormones in form of tightly packed amyloids, releasing monomeric hormone units under determinate stimuli, which would obviate the need of a repeated *de novo* synthesis without affecting the immediate availability of the substance. The further confinement of hormone polymers within a coating membrane provides a means for cells to store amyloid under relatively innocuous conditions.

In spite of the strong pathologic link to disease, it appears that under certain conditions cells are also able to exploit the amyloid properties of prions for functional profit. Major mechanistic insights on prion function derive from the yeast *Saccharomyces cerevisiae*, an organism that ingeniously turns conformational variability underlying prionogenesis into selective advantage (Chernova et al., 2014). In yeast, the insurgence and propagation of new phenotypic traits is sometimes coupled to the ability of determinate proteins to convert from a native into an alternative conformation with a substantially altered original function. This type of conformational rearrangement occurs in a fashion typical of prions, in that the prion conformer (A) displays structural and physical features of amyloids, (B) catalyzes its own template-driven conversion, and (C) can be non-genetically transmitted. As prototypically shown for the yeast protein Sup35 (Tuite and Cox, 2006), a transition from a native [PSI^−^] into a self-perpetuating prion conformation [PSI+] is accompanied by a remarkable acquisition of novel cellular phenotypes, which can be ascribed to a functional loss when soluble Sup35, which is a release factor that controls the fidelity of ribosome translation termination, converts into unsoluble amyloid. Under selective pressure, the functional loss of Sup35 can turn into an advantage, as it improves the prevalence of cryptic phenotypes capable of coping with an altered environment (Halfmann et al., 2012). Even more intriguing, also prokaryotes appear to sustain prion inheritance, being able to propagate the [PSI+] phenotype over several generations under conditions that do not permit *de novo* prion formation (Yuan et al., 2014). This finding points to prion transmission as an ancient mechanism of inheritance.

Although such an impressive strategy shows that prions can be tolerated in principle, bargaining of phenotypic homoeostasis is hardly conceivable in neurons. First, the outspoken toxicity of prions in mammalian neurons overrides any possible adaptation to selective pressure within an evolutionary observable time scale. Second, mammalian cells lack essential regulators of yeast prion inheritance (e.g., the molecular chaperone hsp104).

With this in mind, it might sound heretical to speculate about prion-associated physiological benefits for mammalians. However, also in higher eukaryotes, polypeptides with *bona fide* prion properties exist more frequently than it might sound reasonable for a purely toxic agent. Algorithms trained to identify possible prion signatures could pinpoint one prevailing structural pattern to amino acid stretches of low structural complexity abounding in glutamine and/or asparagine residues. The validity of these predictions could be experimentally verified case-by-case for proteins from yeast as well as from higher eukaryotes (Alberti et al., 2009; Toombs et al., 2010; Couthouis et al., 2011). Interestingly, the isolated region (referred to as prion-like region PLR) is sufficient to confer self-perpetuating traits typical of yeast prions even when artificially fused to unrelated proteins, when interchanged between proteins, or when heterologuosly expressed in yeast (Sondheimer and Lindquist, 2000; Gilks et al., 2004; Li et al., 2014; Udan-Johns et al., 2014).

Such a typically modular architecture of prion-like regions comes along with an autonomous ability to inherently fold into amyloid-like conformations (Kato et al., 2012). Given that protein modules serve for a precise functional purpose, it can be expected that amyloid folding provides more than the structural blueprint of proteotoxicity. For the neuronal isoform of cytoplasmic polyadenylation element-binding protein (CPEB) from the aquatic snail *Aplysia californica*, the aggregation into an active polymer is essential to regulate the simultaneous processing of multiple RNA molecules in a spatially organized and highly coordinated fashion (Raveendra et al., 2013). Amyloid aggregation guides the local and reversible assembly of CPEB monomers at the synaptic ends of neurons, where this protein operates for long-term memory purposes. The resulting supramolecule is functionally active, showing a much higher RNA binding affinity than the monomeric units. Biostructural analyses have confirmed that functional CPEB aggregates display a typical amyloid conformation, consisting of a high beta-sheet content and an x-ray diffraction pattern at 4.7 and 10.7 A. Importantly, the RNA-binding domain does not incorporate into amyloid fibers and remains solvent exposed, substantiating the hypothesis that only the prion-like region functions like an autonomous and specialized structural module.

In analogy, prion-like assembly is a functional aspect of RNA-binding protein FUS, a 53 kDa protein with an aggregation propensity that is typically linked to the occurrence of unsoluble protein inclusions in amyotrophic lateral clerosis (ALS) and frontotemporal dementia (FTD; Shelkovnikova, 2013). In fundamental contrast to such a type of irreversible aggregation, some recent studies point to FUS self-assembly as a reversible, regulated, and explicitly functional process. Schwartz et al. describe one type of RNA-induced FUS polymers as characteristic amyloid beta-zipper structures with a significantly increased affinity for RNA polymerase II (RNAPolII) as compared to monomeric FUS (Schwartz et al., 2013). In a separate study, Kwon et al. suggest a biologically regulated nature of this interaction, showing that amyloid-like hydrogels (see Section Prion-like aggregation as an organizing principle of intracellular granule formation) obtained from isolated FUS-PLR bind to RNAPolII in a mode that can be reversed by phosphorylation (Kwon et al., 2013). Further, by using a FUS-PLR/GAL4 gene reporter construct, the authors highlight how mutations of PLR amino acid repeats [G/S]Y[G/S], which are critical for beta-zipper formation, compromise amyloid-like association and transcriptional activation to the same extent, demonstrating that FUS amyloid polymerization is tightly coupled to function. Moreover, a recent study identifies PLR-mediated self-assembly of FUS as an essential process for chromatin binding and transcriptional activity (Yang et al., 2014). Although the authors do not specify the physical properties of the assemblies, the regulatory character of this biochemical process and mechanistic analogies to the RNAPolII complex legitimate the hypothesis of an amyloid-like polymerization. Consistent with such an assumption, the chromatin-associated oligomerisation step requires the presence of RNA, which might trigger amyloidogenesis as a functional prerequisite.

### Prion-like aggregation as an organizing principle of intracellular granule formation

Intriguingly, both CPEB and FUS are representative RNA-binding proteins, a category of polypeptides ranking unsuspectedly high among predicted prion-like candidates. This finding is significant, because proteins such as FUS are constituents of RNA granules, a species of remarkably dynamic organelles that controls major key steps of RNA metabolism from synthesis to splicing, processing, stabilization and degradation (Thomas et al., 2011). While some of them are constitutive, other can generate *de novo* upon specific needs and disassemble afterwards. Such a remarkable plasticity is favored by the lack of a confining membrane, which allows for an uncomplicated interchange of molecules with the intracellular environment. Underlying this dynamics is the property of RNA-binding proteins to shuttle between separate subcellular compartments, to repartition between various types of RNA granules, and to dissociate from them in probable adaptation to selective cellular demand. Systems approaches on neuronal RNA granules suggest that these types of organelles share only little core protein similarities, supporting the fact that the majority of protein components associates transiently and specific to immediate functional requirements. (Fritzsche et al., 2013).

The frequency by which prion-like protein candidates seem to actively influence function, size, and cell number of RNA granules has led to the assumption that their PLRs might serve as rational protein-protein interfaces that naturally control condensation, growth, integrity and dynamic reorganization of these organelles by means of amyloid polymerization. Strong support comes from the observation that some isolated, recombinant PLRs from RNA granule components can spontaneously condensate *in vitro* into hydrogel-like particles with a fibrous morphology and amyloid-like structural properties (Kato et al., 2012). Unlike mature amyloid fibers from unsoluble inclusions, however, these cell-free assemblies are relatively fragile, and reversibly decompose into monomers already under semi-denaturing conditions. Yet, they are sufficiently stable to *in vitro* emulate morphogenesis and steady-state dynamics of RNA granules with staggering simplicity (Han et al., 2012; Weber and Brangwynne, 2012; Kwon et al., 2013): (A) they originate *via* self-assembly either upon concentration-dependent or template-driven nucleation, (B) they undergo demixing phase separations typical of membrane-free organelles, (C) they are able to heterotypically incorporate or exchange additional RNA granule-associated protein components, (D) they tend to associate preferentially with extended 3’-UTR mRNAs sequences, and (E) they respond to post-translatory modifications in a way that reflects physiologic regulation of RNA granules. These findings highlight the potential of PLR-containing proteins to reproduce major hallmarks of RNA granule biogenesis and function by accessing a polymer state which is morphologically close to amyloid, but completely reversible and therefore devoid of any stable higher order aggregates characteristic of pathologic prions. As these polymers unify basic physical properties of stable amyloids (self-organization, cross-beta structure) with reversibility and a minimal toxic hazard, they are potentially appealing for physiological use. First, the spontaneous polymerization of prion-like components can be initiated only upon an incisive conformational change, e.g., by molecules that act as nucleators. Second, polymerization comes along with a sharp phase transition from liquid to gel-like, which closely resembles physical processes of granule condensation *in vivo* (Dundr, 2012; Weber and Brangwynne, 2012). Third, by retaining the unique cross-beta amyloid structure, amyloid-like polymers allow for a tight polymer stacking, which can be useful when a locally restricted accumulation of proteins up to very high concentrations is biologically required. Fourth, their aggregation/deaggregation is susceptible to biochemical stimuli such as post-translatory modifications, allowing for a physiologically organized dynamics.

In principle, these attributes all comply with physical prerequisites of RNA granule assembly and disassembly, such as nucleation upon demand, steady-state association, and a precise spatial and temporal separation, suggesting mechanistic parallels between cell-free hydrogel aggregation and granule biogenesis *in vivo*. The analysis of stress granule (SG) dynamics provides particular support to this assumption. SG are one type of membraneless organelles that originate transiently in the cytosol upon different forms of cellular challenge, contributing to the arrest of mRNA translation in response to generic insults such as intoxication, UV-irradiation, oxidative stress and heat shock (Kedersha et al., 2013). When homeostasis is restored, these granules dissolve rapidly and completely.

The protein and RNA composition of SG is extremely heterogeneous and highly variable, with a growing number of proteins being identified as regulators. This dynamic constitution reflects a possible ability of SG to individually respond and adapt to various types of stimuli. Significantly, SG reversibly incorporate proteins from multiple stress signaling pathways, therefore becoming actively integrated into the circuitry of separate signaling cascades. Phosphorylation of eukaryotic initiation factor 2a (eIF2a), which represents an integrated response to different stress stimuli and is the best investigated trigger of SG assembly, abolishes translation initiation, causing polysome disruption and the passing over of untranslated mRNA to SG under persisting stress (Anderson and Kedersha, 2008). Upon a restored homeostasis, SG rapidly dissociate, thereby releasing bound mRNA for appropriate processing. Further SG components point to regulation of cell death during stress. The incorporation of the adaptor protein RACK1 into SG inhibits the activation of MTK1, a kinase that acts upstream of the p38/JNK apoptosis pathway (Arimoto et al., 2008). Similarly, upon binding to SG, ROCK1, a component of Rho GTPase signaling cascades, loses the ability to transduce apoptotic stimuli (Tsai and Wei, 2010). Finally, SG sequestration of TORC1, a component of the Target of Rapamycin (TOR) pathway, alters metabolism during nutrient deprivation (Wippich et al., 2013). All these effects can be reversed upon SG dissociation, in alignment with a regulatory nature of these interactions, which leads to ask how the SG proteome can adapt its composition to selected stimuli.

One possible explanation comes from the observation that several SG-associated signaling proteins can couple SG initiation to concomitant pathway-specific protein-protein interactions. As shown for dual specificity tyrosine-phosphorylation-regulated kinase 3 (DYRK3), this enzyme nucleates SG in response to a signal-induced concentration increase *via* an N-terminal low complexity region, which is concurrently required for the sequestration and functional inactivation of TORC1 (Wippich et al., 2013). As overexpressing the isolated N-terminal region is sufficient to simultaneously evoke both processes, it is likely that DYRK3 induces the condensation of granules specifically tailored to fit TOR signaling demands.

Although it is unclear whether DYRK3-mediated SG assembly actually involves amyloid-associated polymerization, it is intriguing to note how the majority of established SG-nucleating proteins have a significantly high predicted amyloidogenic propensity (Table 2). One more explicit indication comes from the study of the two prototypic SG nucleators TIA-1 and TIA-1 related (TIAR). Both proteins possess three distinct RNA binding domains (RRM1-3), and under normal conditions, they absolve splicing-associated roles in the nucleus, where TIA-1 has been described to recognize poly-uridine sequences to facilitate 5’ splice site recognition by U1 small nuclear ribonuclein (Izquierdo et al., 2005; Singh et al., 2011). Under stress conditions, these proteins can sense the increase of translationally stalled mRNA by reversibly binding to AU-rich elements in the 3’-UTR (Kedersha et al., 2000). They subsequently move to the cytosol and nucleate the assembly of SG, a process that requires their intact C-terminal Q/N-rich PLR region, a low structural complexity sequence with a high predicted prion score (Gilks et al., 2004; Couthouis et al., 2011). Accordingly, TIA-1 is capable of self-sustained transmission in yeast (Li et al., 2014), and it can associate into amyloid fibrils *in vitro* (Furukawa et al., 2009; Li et al., 2014). While the deletion of the PLR region completely abolishes SG assembly, its replacement with an extraneous PLR with similar aggregation properties (e.g., that of yeast prion Sup35) completely restores the ability (Gilks et al., 2004), which is a striking indication for prion-like aggregation as an inherent mechanism of SG self-organization. In close conjunction, aggregated TIA-1 acquires the ability of binding stalled ribosomes, suggesting that this protein mediates the efficient sequestration of abortive preinitiation complexes by nucleating SG assembly (Gilks et al., 2004). These data provide a further demonstration of how SG can be modeled in response to specific functional purposes, further suggesting that amyloids can serve as structural backbone and adapter modules of highly dynamic supramolecular machines.

**Table 2:** **Amyloidogenic propensity of SG nucleating proteins (adapted from Kedersha et al., 2013) calculated by the PASTA algorithm, which evaluates the stability of putative cross-beta pairings between different amino acid stretches (Walsh et al., 2014)**.

Protein	Lowest energy value
Ago2	−9.49
Ataxin2	−9.06
Caprin1	−6.49
CPEB	−7.07
DDX3	−8.21
DYRK3	−6.77
FASTK	−10.38
FMR1	−6.94
G3BP1	−6.94
MEX3B	−7.44
PARP1	−6.94
PKR	−6.37
PQBP1	−3.75
DAZAP2	−4.84
Pumilio	−7.70
DHX36	−13.24
Roquin	−10.42
SMAUG	−7.25
SMN	−6.67
TIA1	−6.12
TIAR	−6.13
TTP	−4.9

*The list reports the best aggregation pairing energy values for the most aggregation prone peptides. Values below a threshold of −5 are considered confidential. Values of representative amyloidogenic peptides from PrP, aSyn and Abeta are −16.04; −7.24; and −8.86, respectively*.

### Stress granules misassembly as an inherent risk of functional protein aggregation

Intriguingly, the overexpression of an isolated TIA-1 PLR lacking the three RNA binding domains generates deteriorated aggregates which are no longer reversible, and refractory to SDS denaturation and protease digestion (Gilks et al., 2004), a pattern typical of pathogenic prions (see Section Molecular pathways of amyloid propagation). This type of aggregates is not functional, being unable to recruit ribosomes, and insensitive towards conventional degradation (see Section Molecular catchers in the amyloid rye: heat shock proteins and proteolytic pathways), which leads to an unusual cytosolic persistence (several days instead of a few hours) and precipitation. This process anticipates the catastrophic consequences of an uncontrollable and irreversible aggregation, giving an impressive suggestion of why functional aggregation must imply reversibility. This observation is outstanding, considering that alterations in SG integrity, such as assembly/disassembly disequilibria or the improper incorporation of protein components correlate to the prevalence of organic disorders (Banfield et al., 2014). Indeed, in the cell, many regulatory networks are composed of abundant, but thermodynamically unstable (supersaturated) proteins, which are primarily vulnerable to the appearance of excessively stabilized aggregates (Ciryam et al., 2013). While soluble under homeostatic conditions, this type of proteins can easily tilt into unsoluble upon any unbuffered conformational perturbation (Xu et al., 2013). Olzscha et al. have emulated *in situ* the effects of amyloid overload (Olzscha et al., 2011). The exaggerated exposure of cells to artificially superstabilised amyloid particles caused the collapse of entire metastable protein pathways, affecting RNA metabolism, protein turnover, and mitochondrial integrity among others. All these activities are indeed gravely impaired in amyloid-associated neurodegenerative disorders, and significantly, supersaturated proteins abound within neurodegenerative disease pathways (Ciryam et al., 2013).

This concept can be extended to SG, as these organelles must preserve reversibility as a prerequisite for physiological function (Kedersha et al., 2000). Processes that modify the assembly/disassembly, and thereby the steady-state integrity of SG, have been associated to misfolding, mislocalisation, or sequestration of various SG components, such as TDP-43, FUS, and hnRNPA isoforms, which are all outstanding modifiers of inclusion body neuropathologies (Iguchi et al., 2013; Kim et al., 2013; Shelkovnikova, 2013). TDP-43 is a 43 kDa protein originally identified as a transcriptional repressor of HIV-1 transactivation response element. It is aggregation prone (Johnson et al., 2009), and unsoluble intracellular inclusions containing this molecule can be found in patients suffering from FTD and ALS. This protein seems to absolve multiple RNA-devoted roles and it is predominantly nuclear, associating preferentially with splicing components. In the cytosol, endogenous TDP-43 interacts with components of the translation machinery, and it incorporates into SG upon various acute stress stimuli (arsenite intoxication, heat shock, proteasome inhibition). Although it is not a primary nucleator of SG, this protein influences size, morphology, and on/off kinetics of this organelle. Cells deprived of TDP-43 or mutations that decrease SG incorporation (R361S) lead to a significant delay in the appearance of SG, which are smaller in size and of less regular shape (Colombrita et al., 2009; Liu-Yesucevitz et al., 2010; McDonald et al., 2011).

**Figure 1:**
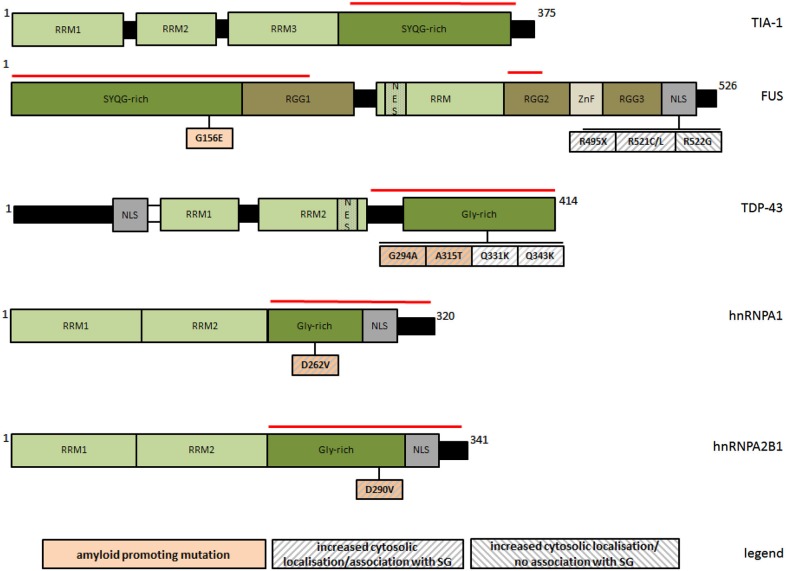
**Schematic representation of PLR-containing SG-associated proteins and of natural mutations discussed in this review with respect to their amyloid and SG-modifying properties**. The red line marks respective prion-like regions. RRM: RNA recognition motif; RGG: glyine/arginine rich domain; NES: nuclear export signal; NLS: nuclear localization signal; ZnF: zinc finger domain; Gly-rich: glycine rich region; SYQG-rich: serine/tyrosine/glutamine/glycine rich region.

While the localization of TDP-43 to SG is reversible and usually restricted to acute stress conditions, a forced cytosolic permanence of TDP-43, either upon persistent stress or due to genetic mutations, seems to potentiate the appearance of irreversible protein aggregates (Liu-Yesucevitz et al., 2010; Bentmann et al., 2012; Parker et al., 2012). In cells exposed to sustained stress, TDP-43 initially localizes to SG, but finally assembles into unsoluble cytoplasmic inclusions resistant to SG-disrupting chemicals such as cycloheximide (Parker et al., 2012). A similar effect was observed after the expression of disease-associated mutants G294A, A315T, Q331K, and Q343R (Figure 1), which all have an increased cytosolic/SG localization pattern (Liu-Yesucevitz et al., 2010) and a major aggregation propensity (Johnson et al., 2009; Chen et al., 2010; Guo et al., 2011). In the presence of these mutants, SG were more abundant upon induction, and in contrast to wildtype-TDP-43, they partially converted into unsoluble species. These findings suggest that under adverse conditions, the incorporation of TDP-43 into SG can favor their conversion from physiologically regulated reversible organelles into irreversible cytosolic aggregates.

Similarly to TDP-43, FUS is an RNA-binding protein with probable roles in transcriptional activation and gene splicing (Schwartz et al., 2012, 2013; Yang et al., 2014), and consistently, it resides predominantly in the nucleus. The association of wildtype FUS with SG seems to be much weaker and more stimulus selective than for TDP-43 (Acosta et al., 2014), but it becomes generally favored upon a forced cytosolic permanence, such as for the disease-associated variants R495X, R521C, R521L, all impairing nuclear localization of FUS (Figure 1). These mutants have an increased aggregation propensity, and readily incorporate into SG upon arsenite treatment (Bosco et al., 2010; Aulas et al., 2012; Bentmann et al., 2012; Baron et al., 2013; Acosta et al., 2014). In analogy to TDP-43, the interaction is still reversible, but with evident alterations in SG morphology and dynamics, as shown by ALS-linked FUS-R945X. This NLS lacking truncation mutant increases the number and size of SG upon persisting stress, although initially disfavoring the assembly of SG (Bosco et al., 2010). Intriguingly, natural or artificial FUS mutants (e.g., R522G) fail to incorporate into SG, accumulating as separate cytosolic inclusions with morphological and dynamic features distinct from SG, and more reminiscent of intracellular bodies associated to neuropatholgic disorders (Shelkovnikova et al., 2014). In contrast to SG, this type of FUS inclusions has a more irregular shape, a much higher kinetic stability than SG, the ability to fuse with each other, and it barely contains SG markers. These findings lead to speculate that SG might transiently incorporate mislocalised and aggregation-prone variants of FUS to prevent aggregation during acute stress, however at the increased risk of an own overload and subsequent precipitation when the stress stimuli become excessive.

Mislocalisation of FUS seems to have multiple pathologic overlaps, as its prolonged extranuclear permanence, while affecting SG dynamics, also prevents this protein from correctly absolving typically nuclear functions. Wildtype FUS can bind to transcriptionally active chromatin, acting as a possible transcriptional regulator (Yang et al., 2014). This property is challenged when nuclear localization is impaired, as for FUS-R495X, which is transcriptionally far less active than the wildytpe. Moreover, mislocalised FUS is no longer able to assist the biogenesis of gems, a class of nuclear RNA processing granules with major roles in spliceosome organization (Yamazaki et al., 2012), leading to a condition closely similar to the motoneuron disorder spinal muscular atrophy (SMA). This type of disease is otherwise linked to mutations in SMN, a protein which is essential for the structural and functional integrity of gems (Liu and Dreyfuss, 1996). It was shown that FUS can interact with SMN, contributing to the regulation of homeostatic gem assembly, a property which was no longer observable for the R495X truncation variant. Therefore, one defect might link the occurrence of cytoplasmic inclusions in ALS with the loss of nuclear gems in SMA, suggesting a converging mechanism for both diseases.

As TDP-43, FUS and hnRNPA are amyloidogenic (Table 1), it is relevant to ask how this property might influence their association with SG. Some more amyloidogenic protein variants exhibit indeed a significantly enhanced tendency to incorporate into SG (Figure 1), suggesting a causal link between both processes. Recently discovered ALS/multisystem proteinopathy-associated hnRNPA1 and hnRNPA2B1 variants (D262V and D290V, respectively) show an increased tendency to form steric zippers, the complementary beta-stranded backbone of amyloid fibers, in concomitance to a significantly more pronounced recruitment to SG (Kim et al., 2013; Figure 1). Moreover, the removal of multiple tyrosine residues at different positions of [G/S]Y[G/S] beta-zipper nucleating repeats of FUS-PLR abolishes both amyloid-like hydrogel polymerization (see Section Prion-like aggregation as an organizing principle of intracellular granule formation) and the incorporation into SG (Kato et al., 2012). Such an explicit relationship has not been demonstrated for TDP-43, although two natural mutations (G294A and A315T) have been separately described as more amyloidogenic (Chen et al., 2010; Guo et al., 2011) and more prone to associate with SG (Liu-Yesucevitz et al., 2010; Figure 1).

SG dynamics is also challenged by amyloidogenic proteins without a “canonical” prion-like region, such as htt or tau. Huntington Disease (HD) key pathogen htt displays a highly variable polyglutamine (polyQ) sequence that develops amyloidogenic and pathogenic properties above a threshold length of around 40 glutamines. In a mouse model for HD, the intracellular aggregation of a 42Q htt variant caused a massive co-aggregation and functional inactivation of TIA-1 (Furukawa et al., 2009). This process is likely to involve cross-nucleation, as htt-derived amyloid fibers were able to seed for TIA-1 amyloids. Intriguingly, non-aggregating htt (25Q) seems to physiologically associate with components of RNA granules without compromising their activity (Savas et al., 2010), suggesting that htt evolves from granule-stabilizing into granule-destabilizing upon the acquisition of amyloidogenic traits. A similar disease-linked association likely occurs for hyperphosphorylated tau, which co-localizes with TIA-1 positive SGs in animal disease models (Vanderweyde et al., 2012). Interestingly, this interaction is dependent on the progression of the disease, being weak to moderate at the beginning, and increasing with disease severity.

Collectively, these data suggest that SG are extremely sensitive to processes affecting their transient nature, with an excessive stabilization leading them to evolve into irreversible toxic aggregates comparable to those of artificially stabilized amyloids (Olzscha et al., 2011). This might explain their rigorously time-, space- and stimulus-confined mode of action.

## The boundaries of tolerated protein aggregation

The previous section has capitalized how functional aggregation occurs incredibly close to catastrophe, and how the boundaries between function and toxicity are startlingly fragile. This leads to wonder what kind of strategies cells adopt to physiologically benefit of a process that goes hand in hand with major system failure.

Gsponer and Babu propose that aggregation can indeed defy proteotoxicity as long as it is controllable, reversible, and temporally and spatially localized (Gsponer and Babu, 2012). They point to the existence of multilevel control mechanisms by which cells manage to keep the concentration of aggregation-prone proteins constitutively below a critical threshold. Aggregation can then be driven by a self-organized monomer-polymer transition following Le-Chatelier’s principle basically by increasing protein abundance upon specific demand. Such a mechanism would apply to explain the dynamic consistency of SG and other transient cellular aggregates, which might form and disrupt upon precise fluctuations of their protein components.

In substance, the fates of aggregation-prone proteins are governed at nucleic acid and protein level. At nucleic acid level, a stringent control of transcripts aims at reducing the expression of aggregation-prone proteins to an essential minimum. The physical regulation of mRNAs encoding aggregation-prone proteins is fundamentally different from mRNAs of non-aggregating proteins. The former type of mRNA has a slower transcription rate, it is preferentially escorted by regulatory RNA-binding proteins, it has a higher tendency to form secondary structure retarding the initiation of translation, and it has mediocre translation efficiency due to a less optimal codon usage and a lower ribosome density per transcript (Gsponer and Babu, 2012).

At protein level, a scrupulous surveillance of protein function and folding quality aims at counteracting the accumulation of misfolded and dysfunctional polypeptides (Hipp et al., 2014). Cells are equipped with high-fidelity machineries that systematically minimize the accumulation of misfolded proteins by coupling protein quality control and clearance. These processes are kept in balance by an intensive cross-talk between molecular chaperones and different degradation machineries. Molecular chaperones are proteins capable of discriminating native from non-native and aggregation-prone protein conformations, selecting irreparably misfolded polypeptides for degradation. This decisional power is fundamental to neutralize adverse effects of irreversibly misfolded proteins which have been rated irrecoverable after failing chaperone quality screening. In this case, substrates are redirected from folding to degradation.

### Molecular catchers in the amyloid rye: heat shock proteins and proteolytic pathways

Heat shock proteins (hsps) are a class of representative and ubi-quitously expressed, structurally unrelated molecular chaperones which bind to unfolded or partially folded polypeptides preventing them from aggregation. Hsps act in frequent combination with each other, thereby providing a powerful relay team, which is equipped with numerous accessory proteins accounting for fine-tuning and coordination.

Two potent modifiers of amyloid fiber assembly are hsp90 and hsp70. While hsp70 family chaperones assist generic protein folding processes (e.g., during polypeptide biosynthesis or membrane translocation), hsp90 acts especially to stabilize proteins in a near-native, yet unstable conformation until their full structural maturation. The rationale behind the stabilization of partially rather than entirely unfolded polypeptides is to locally protect unstable or destabilized regions, e.g., during rearrangements that functionally require the transient exposure of hydrophobic moieties (e.g., during the activation of kinases or steroid hormone receptors). Hsp90 thus prevents the collapse or the aggregation of metastable regions by keeping them in a stalled position (Eckl and Richter, 2013).

As hsp70 and hsp90 influence amyloid fiber assembly at substoichiometric amounts, it has been proposed that a transient interaction occurs already with low-abundance amyloid precursors in order to repartition them from toxic into non-toxic early during amyloidogenesis (Wacker et al., 2004; Evans et al., 2006; Falsone et al., 2009; Daturpalli et al., 2013).

Both chaperones require ATP for functioning, and although the sole presence of nucleotide-free hsp70 and hsp90 is sufficient to suppress amyloid fiber growth *in vitro*, it is only by consumption of ATP that these chaperones actively redirect oligomeric intermediates “on- pathway” for amyloid assembly (Falsone et al., 2009; Lotz et al., 2010). These findings underscore the importance of ATPase-modulating co-factors for a controlled processing of hazardous aggregates. Hsp70 requires a classic cooperation with co-chaperone hsp40 to efficiently neutralize toxic polyQ-htt in an energy-consuming fashion (Lotz et al., 2010). The underlying mechanism is the property of hsp40 to selectively recognize a specific subset of alternatively folded polyQ-htt aggregates originating during the initial amyloid growth lag phase, while leaving other aggregates unaffected. The targeted aggregates are antigenic for the conformational antibody A11, which is selective for “off-pathway” species. These are unable to template for fiber assembly, being most likely toxic. Hsp40 subsequently presents them to hsp70, and the resulting stimulation of the hsp70 ATPase leads to their active remodeling and “on-pathway” redirection for the assembly into less toxic mature fibers.

Several studies highlight the relationships between hsps and amyloid diseases. The deletion of hsp70 genes exacerbates pathogenesis of HD (Wacker et al., 2009), while the overexpression of hsp70 can reduce amyloid-related phenotypes (Klucken et al., 2004). Hsp90 was found in association with polyQ-repeat expansions, showing a high affinity for polyQ-expanded androgen receptor (AR), a pathogenic variant in spinal and bulbar muscular atrophy (Waza et al., 2005). Binding to wild type AR was more transient, and polyQ-AR was preferentially degraded after disrupting this interaction.

The expression of hsp70 and hsp90 is frequently perturbed in neurodegenerative disorders, and both proteins can be recovered from different cytoplasmic inclusions along with other types of chaperones (Hauser et al., 2005). This is consistent with the observation that a persistent exposure to aggregates causes the precipitation of major heat shock proteins, which is equal to a complete failure of protein folding pathways (Olzscha et al., 2011). With respect to SG, hsp90 and hsp70 supervise physiologic assembly of these granules by physically stabilizing separate components (Pare et al., 2009; Udan-Johns et al., 2014), but they are apparently unable to counteract excessive on/off disequilibria, e.g., the accumulation of aberrantly stable TIA-1 aggregates. Unlike full-length TIA-1, fragments from the prion-like region of TIA-1 lead to more stable intracellular aggregates resistant to disaggregation (Gilks et al., 2004; see Section Stress Granules misassembly as an inherent risk of functional protein aggregation). Although the cell reacts by increasing the levels of some endogenous hsps, it is only the artificial overexpression of hsp70 that efficiently reverses the aggregation of TIA-1 prion-like region. Otherwise, the endogenous increase of hsps is not sufficient to impede the formation of intracellular inclusions and the concomitant co-precipitation of hsps. This suggests that chaperones can neutralize acute proteotoxic burden, but not a chronic overload.

In this context, it was found that hsp70 is more inclined to precipitate depending on the state of bound nucleotide (Roodveldt et al., 2009), implying that a functioning nucleotide turnover is essential to guarantee the stability of the cycle. This constitutes a far-reaching challenge, as any stalling of the hsp70 cycle might come along with chaperone deprivation, leading to a derangement of protein folding homeostasis. Generally, such a scenario might be evoked by any form of energetic misbalance, such as mitochondrial impairment, as frequently observed in neurodegeneration (Pathak et al., 2013).

In close relation, pathologically stabilized amyloid species appear to compete with physiological substrates for binding to hsps. As shown for aSyn, stable oligomers can inhibit hsp70, which is therefore no longer capable of functionally folding other substrates (Hinault et al., 2010). Similarly, pathologic tau displaces TDP-43 from cdc37, a dedicated hsp90 co-chaperone. Under normal conditions, hsp90 binds to and stabilizes TDP-43, and the pharmacologic inhibition of hsp90 primes TDP-43 for degradation (Falsone et al., 2007; Zhang et al., 2010). In cooperation, hsp90 and cdc37 regulate TDP-43 turnover and the autophagic clearance of cleaved TDP-43 (Jinwal et al., 2012). However, the inhibition of hsp90/cdc37 activity by hyperphosphorylated tau leads to the cytosolic accumulation of TDP-43 fragments, which eventually precipitate forming inclusions typical of ALS and FTD.

Hsp70 and hsp90 are closely interlaced with protein degradation, and both chaperones possess the ability to redirect toxic aggregates to degradation (Hipp et al., 2014). Consistently, degrading pathways take over misfolded polypeptides which have been sorted for proteolytic clearance. These are essentially the ubiquitin-proteasome system (UPS), chaperone–mediated autophagy (CMA), and macroautophagy (MA). The UPS is constituted of the core protease machinery called 26S proteasome, and a series of enzyme classes controlling substrate recognition, selection and targeting. For proteasome degradation, proteins have to be covalently marked by ubiquitin, a protein that is frequently found within protein inclusions (Dantuma and Bott, 2014). CMA consists in targeting polypeptides to the surface of lysosomes for their specific translocation through the membrane and the subsequent degradation. MA consists in the engulfment of a selected cargo into a double-membrane vesicle, with subsequent fusion to endosomes or directly to lysosomes. All these degradation mechanisms are directly involved in the clearance of amyloid aggregates at different stages. Partially redundant overlaps between proteolytic pathways additionally provide a compensatory advantage, since one malfunctioning complex can be usually backed by the mutual activation of at least another complex, keeping the toxic burden innocuously low. Such a fail-safe strategy is very effective at intransigently counteracting the risks arising from protein misfolding. Indeed, the entire amyloid folding landscape is rigorously subjected to this type of control. With functional aggregation occurring within supervised boundaries, pathogenic drifts towards misfolding can be timely suppressed and potentially toxic aggregates can be delivered to the most qualified clearance system. By these means, the cell can broadly manage the turnover of different physical species of protein aggregates in the most convenient way (Wang et al., 2009; Ebrahimi-Fakhari et al., 2011; Koga et al., 2011; Scotter et al., 2014). The UPS controls the homeostatic turnover of single monomeric proteins, whereas larger soluble or unsoluble aggregates require bulk autophagy. Therefore, proteins that aggregate in consequence to a single-molecule degradation failure can be removed by autophagy depending on size, morphology, and stability. Collectively, these systems provide a regulatory basis for functional aggregation, as they can reversibly shift monomer-oligomer transitions by regulating the turnover, and therefore the effective cellular concentration of each component. The aggregation of TDP-43, for example, can be kept in physiologic equilibrium by the interplay of UPS and autophagy, whereby monomeric TDP-43 can be degraded by the proteasome and soluble oligomers become targeted by MA (Scotter et al., 2014).

UPS degradation of soluble monomers seems to prevail under homeostatic conditions, and consistently, amyloidogenic proteins are substrates of various E3 ubiquitin ligases (Kumar et al., 2012). For aSyn, the degree of ubiquitylation affects amyloid aggregation in a way that reminds of active amyloid repartitioning preceding proteolytic clearance (Haj-Yahya et al., 2013), with ubiquitin chain length regulating stability, aggregation, phosphorylation, and clearance.

While ubiquitylation usually occurs on internal lysine residues, some disease-associated fragments from tau, aSyn, TDP-43 and Abeta can also undergo an unusual N-terminal ubiquitylation (Brower et al., 2013). This form of modification, known as N-end rule pathway, consists of polyubiquitylation of N-terminal arginine residues by specific E3 ligases called N-recognins. Upon their conjugation, each of these fragments undergoes rapid proteasomal degradation, which impedes an excessive accumulation of these highly aggregation-prone and proteopathic species. For Abeta fragments, an additional N-terminal argininylation configures these peptides for the subsequent ubiquitin conjugation step.

These findings highlight the predominant role of ubiquitin-mediated degradation in neuroprotection. Proteasome dysfunction seems central to neurodegeneration, and rather generic impairments of the UPS, whether pharmacologic, genetic, or physiologic, are sufficient for the unbiased appearance of diverse neurodegenerative phenotypes. The chemical UPS inhibition of dopaminergic cells favors the accumulation of Lewy-body like inclusions (Rideout et al., 2001). Likewise, the stereotactic injection of proteasome inhibitors into mice brains causes symptoms closely related to PD, including dopaminergic neuronal death, decreased motor activities, and the accumulation of inclusions positive to aSyn and ubiquitin (Xie et al., 2010). Moreover, the deletion of single proteasomal (but not autophagic) subunits is sufficient to evoke ALS symptoms in knock-out mice, leading to mislocalisation and precipitation of TDP-43 and FUS in motor neurons (Tashiro et al., 2012).

The integrity of the proteasome can be further challenged by an uncontrolled overload of amyloid aggregates. For Abeta, it was shown that aggregates can overturn the function of the UPS by physically interacting with proteasome subunits. Zhao and Yang suggest that a decreased proteasome activity is not due to inhibition, but rather to the competition of natural proteasome substrates with increasing concentrations of amyloid aggregates, as shown for Abeta peptide (Zhao and Yang, 2010). Similarly, the intracellular accumulation of TDP-43 or aSyn aggregates comes along with massive UPS dysfunction (Nonaka et al., 2013; Tanik et al., 2013). These considerations underscore the importance of a compensatory cross-talk between each single proteolytic pathway, whereby stimulating one separate degradation machinery can efficiently rescue malfunctions of the other pathways (Xilouri et al., 2013). By these means, aggregates that become renitent to conventional disposal can be passed over for alternative clearance. aSyn, which is usually cleared by the UPS under normal conditions, can be passed over to MA during particular cellular challenges (Ebrahimi-Fakhari et al., 2011). In HD, a failed segregation of cytosolic cargo correlates with a marked increase of CMA components (Koga et al., 2011). An effect in the opposite direction is observed for disease-linked tau fragments, as the failure to process them via CMA leads to the activation of MA (Wang et al., 2009).

While the folding/degradation machineries presented above represent major aggregate modifying pathways, the recent discovery of the protein class MOAG-4/SERF suggests also the existence of more selective cellular mechanisms. MOAG-4/SERF possesses the unique ability to distinguish between amyloid and non-amyloid/amorphous aggregation, acting as a potent amyloid promoting factor (Falsone et al., 2012). One possible mechanistic interpretation of this property comes from the observation that the human homologue SERF1 binds to the C-terminal region of aSyn, which usually shields the central amyloidogenic region of this protein (Dedmon et al., 2005). The exposure of amyloidogenic core regions would thereby facilitate amyloid self-assembly. Intriguingly, the absence/presence of MOAG/SERF has pronounced effects on the proteotoxicity of intracellular htt, Abeta and aSyn aggregates in different model organisms. In* C. elegans*, aggregation and toxicity of polyQ expansions change in relation to the ageing-dependent expression levels of the orthologue MOAG-4 (van Ham et al., 2010). Silencing of MOAG-4 leads to a suppression of polyQ aggregation and toxicity, while the overexpression significantly aggravates toxicity, shifting polyQ towards compact misfolding intermediates. This effect is independent of conventional amyloid modifying pathways, as it is not influenced by alterations of the heat shock response, the UPS, or autophagy.

At this time, a more defined functional classification of MOAG/SERF is precluded by to the lack of detailed functional data. Of note, human homologues SERF1 and SERF2 share low-level homology with the RNA-binding domain of Matrin3 (Scharf et al., 1998) a protein that colocalises with small nuclear ribonucleoproteins (snRNPs), interacts with TDP-43, and has been recently identified as a novel disease marker for some rare forms of ALS (Johnson et al., 2014). This might suggest a possible role in RNA-associated (pathologic) processes.

## Concluding remarks

The purely pathological significance of amyloid protein aggregation has been questioned by the discovery of physiological processes that exploit some aspects of amyloid polymerization apparently for functional purposes. The structural and mechanistic similarity between pathologic and functional particles, however, anticipates that benefits of amyloid aggregation go hand in hand with toxicity. Despite these imminent hazards, the cell seems to tolerate aggregation as long as strictly confined within spatially and temporally delimited boundaries, as defined by inherently low transcription and translation rates, localized changes in protein levels, and a high protein quality control and turnover. By these means, aggregation-prone polypeptides are subjected to an exceptional physical and biological supervision at almost every level of cellular life. Yet, surveillance systems can fail upon chronic exposure to abnormally resistant aggregates, and in spite of some considerable functional backup, multiple simultaneous dysfunctions can tilt the equilibrium from functional into toxic. It is therefore conceivable, that amyloid polymerization is physiological, as long as the cell is capable of buffering any inherent toxicity. The occurrence of multiple system failures, as for example during cell senescence or upon extraordinary cellular challenges, might lead to the prevalence of toxic phenotypes, reflecting the multifactorial nature of the associated clinical conditions.

## Conflict of interest statement

The authors declare that the research was conducted in the absence of any commercial or financial relationships that could be construed as a potential conflict of interest.

## Abbreviations

CNS: central nervous system
PD: Parkinson Disease
AD: Alzheimer Disease
ALS: amyotrophic lateral sclerosis
FTD: frontotemporal dementia
HD: Huntington Disease
aSyn: alpha-synuclein
Abeta: beta-amyloid
prp: major prion protein
htt: huntingtin
PLR: prion-like region
SG: stress granules
hsp: heat shock protein
UPS: ubiquitin-proteasome system
CMA: chaperone–mediated autophagy
MA: macroautophagy
GAG: glycosaminoglycan
HSPG: heparan suphate proteoglycans.
